# Characterization of Context-Dependent Effects on Synthetic Promoters

**DOI:** 10.3389/fbioe.2020.00551

**Published:** 2020-06-12

**Authors:** Sebastian Köbbing, Lars M. Blank, Nick Wierckx

**Affiliations:** ^1^Institute of Applied Microbiology - iAMB, Aachen Biology and Biotechnology – ABBt, RWTH Aachen University, Aachen, Germany; ^2^Institute of Bio- and Geosciences (IBG-1: Biotechnology), Forschungszentrum Jülich GmbH, Jülich, Germany

**Keywords:** *Pseudomonas putida*, synthetic biology, synthetic promoter libraries, Tn7 transposon, tandem promoter, heterologous expression

## Abstract

Understanding the composability of genetic elements is central to synthetic biology. Even for seemingly well-known elements such as a sigma 70 promoter the genetic context-dependent variability of promoter activity remains poorly understood. The lack of understanding of sequence to function results in highly limited *de novo* design of novel genetic element combinations. To address this issue, we characterized in detail concatenated “stacked” synthetic promoters including varying spacer sequence lengths and compared the transcription strength to the output of the individual promoters. The proxy for promoter activity, the msfGFP synthesis from stacked promoters was consistently lower than expected from the sum of the activities of the single promoters. While the spacer sequence itself had no activity, it drastically affected promoter activities when placed up- or downstream of a promoter. Single promoter-spacer combinations revealed a bivalent effect on msfGFP synthesis. By systematic analysis of promoter and spacer combinations, a semi-empirical correlation was developed to determine the combined activity of stacked promoters.

## Introduction

The Pseudomonads are a promising group of bacteria for industrial applications (Wierckx et al., 2005; Tiso et al., 2014; Aparicio et al., 2018). A versatile metabolism enables them to grow on several carbon sources like glucose and glycerol, but also on a wide range of aliphatics and aromatics (Jiménez et al., 2002; Nikel et al., 2014; Köhler et al., 2015). Different *Pseudomonas* strains have been engineered for the production of chemicals with industrial importance from different renewable carbon sources, like furandicarboxylic acid, rhamnolipids, and aromatics (Wierckx et al., 2005; Sun et al., 2007; Blank et al., 2008; Koopman et al., 2010; Meijnen et al., 2011; Wynands et al., 2018). *Pseudomonas* is highly tolerant to chemical stresses and can survive harmful conditions caused by oxidative stress (Isken and de Bont, 1998; Ramos et al., 2002; Wierckx et al., 2005; Wynands et al., 2018). Some strains can thrive under a second phase of toxic hydrophobic solvents such as toluene or styrene (Heipieper et al., 2007; Kusumawardhani et al., 2018). *P. putida* KT2440 is a non-pathogenic representative of this versatile group of bacteria (Nelson et al., 2002). The strain is able to produce and accumulate polyhydroxyalkanoates (PHA) as a storage polymer in granules under nitrogen depletion from different carbon sources like glycerol, glucose, ethylene glycol, 1,4-butanediol, or fatty acids (Sun et al., 2007; Wang and Nomura, 2010; Franden et al., 2018; Li et al., 2019, 2020).

Parallel to the increasing industrial interest in Pseudomonads, an ever-increasing set of synthetic biology tools is developed for this genus. These include genomic integration tools like transposon Tn5 (de Lorenzo et al., 1990; Herrero et al., 1990; Nikel and de Lorenzo, 2013) or Tn7 (Lambertsen et al., 2004; Damron et al., 2013; Silva-Rocha and de Lorenzo, 2014), as well as a suite of tools for targeted and marker-less integration (Martínez-García and de Lorenzo, 2011). To obtain gene replacements, counter-selection procedures were established, like *sacB* originating from *Bacillus subtilis* (Schweizer, 1992). New tools are based on CRISPR/Cas9 showing high potential for whole-genome engineering approaches (Jiang et al., 2013; Aparicio et al., 2018). These tools enable a deep genetic and metabolic re-factoring of different Pseudomonads as exemplified in the engineering of streamlined chassis strains (Shen et al., 2017; Wynands et al., 2018; Sánchez-Pascuala et al., 2019).

Especially when such deep engineering entails the (over-) expression of many homologous or heterologous genes, balanced and reliable gene expression is required, which doesn't unnecessarily burden the cell. In this context, calibrated synthetic promoter libraries enable modulation of enzyme expression in metabolic pathways and protein production (Rud et al., 2006; Solem et al., 2007). Two major ways to generate a promoter library are prominently used. A low degeneracy approach, where only a few random nucleotides are introduced, reduces the number of possible generated promoter sequences and thus decreases the number of sequences, which have to be tested (Mutalik et al., 2013). This allows a deeper insight into promoter sequence-activity relationships. On the other hand, high degeneracy promoter libraries based on a degenerated core promoter sequence lead to billions of different possibilities (Zobel et al., 2015; Gilman and Love, 2016; Elmore et al., 2017). While the sequence space clearly outnumbers the experimental space possible to address, a high resolution of different promoter activities is possible. The use of calibrated and standardized synthetic promoters covering a range of activities are commonly used (Zobel et al., 2015). Constitutive synthetic promoters are generally based on sigma-70 (σ^70^) factor core promoters (Gruber and Gross, 2003). The σ^70^ factor encoded by *rpoD* guides the RNA polymerase to many promoters active during growth including the expression of housekeeping genes (Kang et al., 1997; Potvin et al., 2008). Varying the consensus sequences of the −10 and −35 elements, which are recognized by the holoenzyme as part of the core promotor (Lodge et al., 1990; McLean et al., 1997), leads to weaker expression strength in *E. coli* and *P. aeruginosa* (McLean et al., 1997). The σ^70^ factors consensus sequence of *P. putida* KT2440 and *P. aeruginosa* are identical (McLean et al., 1997; Zobel et al., 2015).

Characterization of synthetic promoters has been performed using plasmid-based expression systems or genomically integrated probes (Jensen and Hammer, 1998; Hammer et al., 2006; Zobel et al., 2015). However, varying plasmid copy numbers and high fitness costs for the host makes plasmid-based expression systems less suitable for promoter characterization in particular, and for metabolic engineering in general (Gao et al., 2014; Jahn et al., 2014; Lindmeyer et al., 2015; San Millan and MacLean, 2017). Genomic integration of the probe is preferred for characterization procedures (Zobel et al., 2015). The major difference is the fact that many of the plasmids used are multicopy, which increases the variability of the reporter output by copy number variations. In addition, an often-overlooked disadvantage of using multicopy plasmids for synthetic promoter screening is that they favor the selection of relatively weak promoters, as the combined effect of a strong constitutive promoter at high copy number may pose a too high burden. Genomic integration abolishes these copy number effects, as well as clonal variations, which have also been observed for different *Pseudomonas* strains (Friehs, 2004; Gao et al., 2014; Zobel et al., 2015). Nevertheless, the integration site in the genome must be chosen wisely and must be the same for all promoters. The expression activity differs not only for single genes, but also in larger regions on the genome (“hot” and “cold” spots). Therefore, we used a mini Tn7 transposon, which integrates in a targeted manner downstream of the *glmS* gene in the *attTn7* site of a broad range of bacteria including *P. putida* KT2440, thereby enabling reliable and stable expression (Lambertsen et al., 2004; Choi et al., 2005; Zobel et al., 2015).

Synthetic promoter libraries are described for *P. putida* KT2440 (Zobel et al., 2015; Elmore et al., 2017). However, the predictability and composability of these promoters in different genetic contexts is poorly understood. Li et al. (2012) has shown that different numbers of promoters in tandem direction result in increased activities. Several other publications feature tandem promoters, but so far without a characterization of these promoter combinations that focusses on composability and predictability of the activity of these genetic elements (Dixon, 1984; Martens et al., 2004; Tamsir et al., 2011). The combination of promoters in different contexts is also a key element in logic gate construction in synthetic biology, and sensitivity to genetic context is considered a challenge there (Stanton et al., 2013).

In this work, we stacked (concatenated) promoters of a previously published promoter library from Zobel et al. (2015) in series and analyzed the resulting activities as single genomically integrated probes by measuring *msfGFP* expression (Landgraf, 2012). The obvious assumption that the combination of two promoters would yield their summed activity proved to be false. The reasons for this are investigated and a semi-empirical correlation was developed to reliably predict stacked synthetic promoter activities. This provided insights into the context-dependent activity of promoters that may foster a better predictability and composability of this key element of synthetic biology.

## Materials and Methods

### Bacterial Strains, Plasmids, and Cultivation Conditions

Strains and plasmids used and generated in this study are listed in Table 1. For cloning chemically competent *E. coli* PIR2 (Life Technologies, Carlsbad, USA) were used (Hanahan, 1983). Cultivation of *E. coli* was performed in lysogeny broth (LB) with 5 g L^−1^ NaCl (Sambrook et al., 1989). For solid media 15 g L^−1^ agar was added to the medium before autoclaving. To maintain the mini Tn7 plasmid in *E. coli*, 50 mg L^−1^ kanamycin was added to either liquid or solid medium. For pRK600 (Keen et al., 1988) bearing strains chloramphenicol (10 mg L^−1^) and for pTnS-1 (Choi et al., 2005) ampicillin (100 mg L^−1^) was used. Cultivation of *E. coli* strains was carried out at 37°C. Integration of the mini Tn7 transposon was performed by patch mating on LB agar plates and subsequent cultivation overnight at 30°C (Zobel et al., 2015). This was done with a mini Tn7 suicide plasmid-bearing donor strain, acceptor strain *P. putida* KT2440, and two helper-strains. *E. coli* HB101 (Boyer and Roulland-Dussoix, 1969) bearing plasmid pRK600 (Keen et al., 1988) with mobilization genes. *E. coli* DH5αλpir bearing pTnS-1 (Choi et al., 2005) encodes a transposase for transposition of the mini Tn7 transposon. For selection and counter-selection of Tn7-bearing *P. putida* KT2440, cetrimide agar plates containing 30 mg L^−1^ gentamycin and 1 % glycerol were used.

**Table 1:** Strains and plasmids used and generated in this study.

**Strain**	**Description**	**References**
*E. coli*		
HB101	*F^−^ mcrB mrr hsdS20*(*rB^−^ mB^−^*) *recA13 leuB6 ara-14 proA2 lacY1 galK2 xyl-5 mtl-1 rpsL20*(Sm^R^) *gln V44λ^−^*	Boyer and Roulland-Dussoix (1969)
CC118λpir	Δ(*ara*-*leu*) *araD* Δ*lacX74 galE galK phoA20 thi-1 rpsE rpoB argE*(*Am*) *recA1*, lysogenized with λpir phage	Herrero et al. (1990)
PIR2	F^−^Δ*lac169 rpoS* (Am) *robA1 creC510 hsdR514 endA reacA1 uidA* (Δ*Mlui*)::*pir*	Life Technologies
*E. coli* DH5αλ pir	*endA1 hsdR17 glnV44* (= *supE44*) *thi-1 recA1 gyrA96 relA1* ϕ*80dlac*Δ(*lacZ*)*M15* Δ(*lacZYA*-*argF*)*U169 zdg*-*232*::Tn10 *uidA*::*pir*+	de Lorenzo lab
*P. putida*		
KT2440	Wild-type strain derived of *P. putida* mt-2 cured of the pWW0 plasmid	Bagdasarian et al. (1981)
BG	Gm^R^, *P. putida* KT2440 with genomic insertion of pBG	Zobel et al. (2015)
BG13	Gm^R^, *P. putida* KT2440 with genomic insertion of pBG13	Zobel et al. (2015)
BG14a	Gm^R^, *P. putida* KT2440 with genomic insertion of pBG14a	Zobel et al. (2015)
BG14b	Gm^R^, *P. putida* KT2440 with genomic insertion of pBG14b	Zobel et al. (2015)
BG14c	Gm^R^, *P. putida* KT2440 with genomic insertion of pBG14c	Zobel et al. (2015)
BG14d	Gm^R^, *P. putida* KT2440 with genomic insertion of pBG14d	Zobel et al. (2015)
BG14e	Gm^R^, *P. putida* KT2440 with genomic insertion of pBG14e	Zobel et al. (2015)
BG14f	Gm^R^, *P. putida* KT2440 with genomic insertion of pBG14f	Zobel et al. (2015)
BG14g	Gm^R^, *P. putida* KT2440 with genomic insertion of pBG14g	Zobel et al. (2015)
BG14f_##_14g	Gm^R^, *P. putida* KT2440 with genomic insertion of pBG14f_##_14g, spacer with varying length from ten to 100 bp	This work
BG_80i	Gm^R^, *P. putida* KT2440 with genomic insertion of pBG_80i	This work
BG_80new	Gm^R^, *P. putida* KT2440 with genomic insertion of pBG_80new	This work
BG14x_80i_14y	Gm^R^, *P. putida* KT2440 with genomic insertion of pBG14x_80i_14y	This work
BG14f_80i_14f_80i_14g	Gm^R^, *P. putida* KT2440 with genomic insertion of pBG14f_80i_14f_80i_14g	This work
BG14x_80i	Gm^R^, *P. putida* KT2440 with genomic insertion of pBG14x_80i	This work
BG_80i_14y	Gm^R^, *P. putida* KT2440 with genomic insertion of pBG_80i_14y	This work
BG14f_80new	Gm^R^, *P. putida* KT2440 with genomic insertion of pBG14f_80new	This work
BG_80new_14g	Gm^R^, *P. putida* KT2440 with genomic insertion of pBG_80new_14g	This work
BG14f_80new_14g	Gm^R^, *P. putida* KT2440 with genomic insertion of pBG14f_80new_14g	This work
BG14g_SNP_PosZZn	Gm^R^, *P. putida* KT2440 with genomic insertion of pBG14g_SNP_PosZZ_N	This work
BG14g_PosZZn_80i	Gm^R^, *P. putida* KT2440 with genomic insertion of pBG14f_PosZZ_N_80i	This work
Plasmids		
pRK600	Cm^R^, oriColE1, *tra* + *mob* + of RK2	Keen et al. (1988)
pTnS-1	Ap^R^, oriR6K, *TnSABC*+*D* operon	Choi et al. (2005)
pBG	Km^R^, Gm^R^, oriR6K, Tn7L and Tn7R extremes, BCD2–*msfgfp* fusion	Zobel et al. (2015)
pBG13	Km^R^, Gm^R^, oriR6K, pBG-derived, promoter P_em7_	Martínez-García et al. (2015)
pBG14a	Km^R^, Gm^R^, oriR6K, pBG-derived, promoter 14a	Zobel et al. (2015)
pBG14b	Km^R^, Gm^R^, oriR6K, pBG-derived, promoter 14b	Zobel et al. (2015)
pBG14c	Km^R^, Gm^R^, oriR6K, pBG-derived, promoter 14c	Zobel et al. (2015)
pBG14d	Km^R^, Gm^R^, oriR6K, pBG-derived, promoter 14d	Zobel et al. (2015)
pBG14e	Km^R^, Gm^R^, oriR6K, pBG-derived, promoter 14e	Zobel et al. (2015)
pBG14f	Km^R^, Gm^R^, oriR6K, pBG-derived, promoter 14f	Zobel et al. (2015)
pBG14g	Km^R^, Gm^R^, oriR6K, pBG-derived, promoter 14g	Zobel et al. (2015)
pBG14f_##_14g	Km^R^, Gm^R^, oriR6K, pBG-derived, stacked promoter 14f/14g, spacer with varying length from ten to 100 bp	This work
pBG_80i	Km^R^, Gm^R^, oriR6K, pBG-derived, promoter-less control, reverse complement spacer sequence with 80 bp length	This work
pBG_80new	Km^R^, Gm^R^, oriR6K, pBG-derived, promoter-less control, new spacer sequence with 80 bp length	This work
pBG14x_80i_14y	Km^R^, Gm^R^, oriR6K, pBG-derived, stacked promoter 14x/14y, inverted spacer with a length of 80 bp	This work
pBG14f_80new_14g	Km^R^, Gm^R^, oriR6K, pBG-derived, stacked promoter 14f/14g, new spacer sequence with 80 bp length	This work
pBG14f_80i_14f_80i_14g	Km^R^, Gm^R^, oriR6K, pBG-derived, stacked promoter 14f_80i_14f_80i_14g, inverted spacer with a length of 80 bp	This work
pBG14x_80i	Km^R^, Gm^R^, oriR6K, pBG-derived, first position promoter control 14x, inverted spacer with a length of 80 bp	This work
pBG_80i_14y	Km^R^, Gm^R^, oriR6K, pBG-derived, second position promoter control 14y, inverted spacer with a length of 80 bp	This work
pBG14f_80new	Km^R^, Gm^R^, oriR6K, pBG-derived, first position promoter control 14f, new spacer sequence with 80 bp length	This work
pBG_80bp_14g_new	Km^R^, Gm^R^, oriR6K, pBG-derived, second position promoter control 14g, new spacer sequence with 80 bp length	This work
pBG14g_SNP_PosZZn	Km^R^, Gm^R^, oriR6K, pBG-derived, single nucleotide promoter library with specific positions changes, library is based on 14g	This work
pBG14g_PosZZn_80i	Km^R^, Gm^R^, oriR6K, pBG-derived, first position promoter control 14g_PosZZ_N with modified nucleotide at distinct position of 14g core promoter sequence, inverted spacer with a length of 80 bp	This work

*A complete list containing all strains described more in detail can be found in the Supplementary Table 8*.

##*Distance between two promoters in bp*.

*x stands for promoter 14a to 14g at the first position*.

*y stands for promoter 14a to 14g at the second position*.

*ZZ stands for position 1 to 30 of 14g based SNP*.

*n stands for nucleotides A, C, G, or T*.

Cultivations of *P. putida* KT2440 derivatives for promoter characterization were performed in minimal medium containing 3.88 g L^−1^ K_2_HPO_4_ and 1.63 g L^−1^ NaH_2_PO_4_ with 20 mM glucose as the sole carbon source (Hartmans et al., 1989).

### DNA Techniques

**Figure 1:**
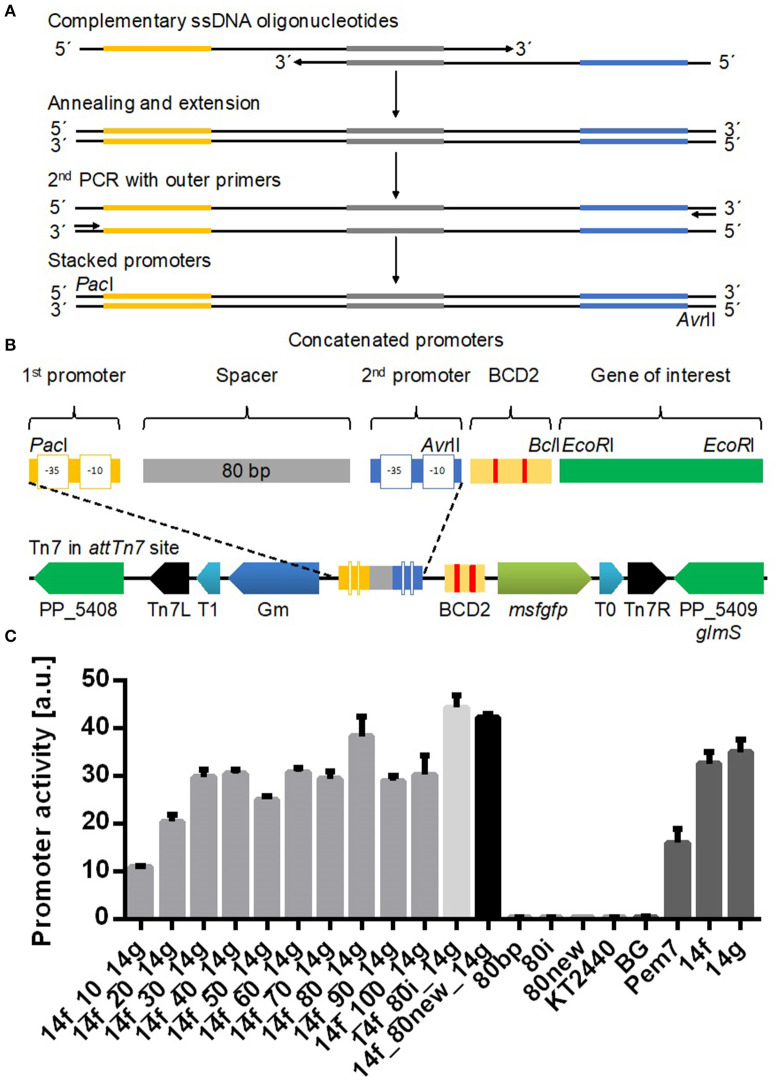
Identification of the optimal spacer length between the two promoters 14f and 14g using a mini Tn7 vector (Zobel et al., 2015). **(A)** Two PCR reactions were performed to generate stacked promoters with longer (>45 bp) spacer sequences and promoter combinations with the 80i spacer sequence. In a first PCR reaction long single stranded DNA oligonucleotides containing one promoter sequence (yellow or blue) were annealed via complementary sequences in the spacer (gray) and extended by Q5 polymerase to double stranded DNA. The resulting dsDNA fragment was amplified in a second PCR. **(B)** Structural organization of stacked promoters and of the mini Tn7 used in this study after genomic integration. Stacked promoters consisting of promoter sequences at two positions separated by a spacer were inserted via restriction sites *Pac*I and *Avr*II. BCD2 element for translational coupling and *msfGFP* as reporter gene. Tn7 module contains a GmR marker for selection and two terminators (T0 and T1) for insulation of the probe. Tn7R and Tn7L are recognized by a transposase. **(C)** Tested spacers contained between 10 and 100 bp. *P. putida* KT2440 *attTn7::*BGf##g-mfsGFP, where ## refers to the number of nucleotides in the spacer sequence (gray bars), were cultured in a BioLector in minimal medium with 20 mM glucose in a 96 well plate. The control strains BG13 with the Pem7 promoter of average strength, the individual promoters 14f and 14g, and promoterless BG and wild type *P. putida* KT2440, as well as additional controls with two 80 bp spacers 80i and 80new are also shown. Identical strains from at least two different transformations were tested, with three biological replicates each. Error bars indicate the standard error of the mean (*n* > 6).

All oligonucleotides used in this study are listed in Supplementary Table 1. For the generation of different length spacer sequences PCR was used (Supplementary Table 2). Up to 40 bp length a forward oligonucleotide (SK11, SK34, SK36, or SK38) and reverse oligonucleotide SK2 were used with plasmid pBG14g as template (Supplementary Table 3). Q5 polymerase (New England Biolabs) with proofreading activity was used for amplification and reactions were prepared as described by the manufacturer. The resulting fragments were cut out from agarose gels and purified with the DNA Gel Extraction kit from New England Biolabs. Longer spacer sequences from 50 to 100 bp were assembled by PCR with two long oligonucleotides with complementary 3′-ends and an initial annealing step in the PCR (Figure 1, Supplementary Table 4). Dimer formation of 3′-ends was checked *in silico* to ensure that stacked promoter constructs can be formed by annealing of two oligonucleotides (https://www.thermofisher.com/de/de/home/brands/thermo-scientific/molecular-biology/molecular-biology-learning-center/molecular-biology-resource-library/thermo-scientific-web-tools/multiple-primer-analyzer.html). A detailed approach is described in the Supporting Information. PCR fragments were purified with PCR & DNA Cleanup Kit (New England Biolabs). Promoter-less plasmid pBG was used as backbone. Generated spacer fragments were digested by *Pac*I and *Avr*II (New England Biolabs) at 37°C. Cloning procedures are following the rules of SEVA and are thus compatible with other constructs (Silva-Rocha et al., 2013). Plasmid pBG was additionally treated with alkaline phosphatase (Fermentas) to circumvent self-ligation. Digested fragments were purified using PCR & DNA Cleanup Kit (New England Biolabs). DNA concentrations of eluted DNA were measured with a NanoDrop One (Thermo Scientific). Fragments and backbone with adjusted concentrations were ligated using T4 ligase (New England Biolabs) at room temperature for 30 min. Transformation of ligated plasmids was performed by heat shock into chemically competent *E. coli* PIR2 cells (Hanahan, 1983). Plasmid isolation was done with Plasmid Miniprep Kit (New England Biolabs) and sequences were confirmed by Sanger sequencing (Eurofins Genomics). Oligonucleotide combinations and detailed PCR protocols are described in Supplementary Table 5.

For the construction of promoter positions and spacer controls, previously cloned plasmids containing stacked promoters were used as template (Supplementary Tables 6, 7).

Construction of the single nucleotide polymorphism (SNP) library was done by PCR with plasmid pBG14g as template and primers SK63-SK92 containing single degenerate nucleotides.

We used colony PCR with oligonucleotides SK4 and SK5 to verify the correct and full-length genomic integration of Tn7 at the *attTn7* site in *P. putida* KT2440. Single colonies were picked with a pipette tip or toothpick and lysed in 30 μL lysis buffer containing 60 % alkaline PEG 200 (pH adjusted to 13–13.5 with 2M KOH) for 15 min at room temperature (Wynands et al., 2018). As template one microliter was used for the PCR reaction (*Taq* 2X Master Mix, New England BioLabs).

### Measuring Fluorescence and Determination of Promoter Activity

For the identification of SNPs in the obtained promoter library for the individual positions we measured GFP fluorescence. We cultivated *E. coli* PIR2 mini Tn7 plasmid-bearing strains in 0.5 mL LB medium containing 50 mg L^−1^ kanamycin at 30°C in 96 well System Duetz plates (Enzyscreen, The Netherlands). Fluorescence of msfGFP was measured in a synergyMX plate reader (Biotek, Bad Friedrichshall, Germany). Samples were measured in black bottom 96 well plates at an excitation wavelength of 488 nm and emission wavelength of 520 nm. Absorption was measured at 600 nm in clear bottom 96 well plates. From strains showing different intensities for GFP fluorescence the plasmid was isolated and sequenced. Followed by genomic integration of desired plasmids in *P. putida* KT2440 by triparental mating.

Growth and fluorescence measurements of integrated promoter constructs in *P. putida* KT2440 were performed with a Biolector (M2P Labs, Baesweiler, Germany) in 96 well plates (Greiner Bio-One) with a filling volume of 200 μL. Cultures were inoculated to an optical density at 600 nm of 0.1 for each strain from precultures cultivated at 30 °C at 300 rpm in 24 well System Duetz plates (Enzyscreen, Heemstede, The Netherlands) containing 1.5 mL of previously described minimal medium. The Biolector was set to 30°C, 900 rpm and humidity control of 85 %. Two internal filter modules of the device were used for online measurement. Fluorescence of GFP was measured at excitation wavelength at 488 nm and emission wavelength of 520 nm with gain 50. Biomass was determined at 620 nm with gain 40 as scattered light. Scattered light was correlated to OD600 with a dilution series of a stationary phase culture. Determination of promoter activity was done with Microsoft Excel by calculating the slope of GFP fluorescence to optical density during the exponential phase.

### Determination of Transcript Levels by Quantitative Real Time PCR

Transcription levels of *msfGFP* was determined by quantitative real time PCR. RNA was isolated from chosen strains grown on minimal medium containing 20 mM glucose as sole carbon source in 24 well System Duetz plates at 30°C and 300 rpm (Hartmans et al., 1989). Biological duplicates of each strain were cultivated until an optical density of 1.0 was reached. One milliliter of cell cultures were harvested, supernatant discarded and the resulting pellet resuspended with 1 mL RNA*later*^TM^ Stabilization Solution (ThermoFisher Scientific). Afterwards the cells were resuspended in 700 μL lyse solution (New England Biolabs, Monarch™ Total RNA Miniprep Kit) and transferred to bead beating tubes containing glass bead with a size of 0.5 mm (Zymo Research, Irvine, USA). Tubes were beaten for 1 min to destroy the cells (Mini-Beadbeater-16, Biospec Products, Bartlesville, USA). Cells debris were removed by centrifugation at 13.000 rpm for 2 min. The supernatant was transferred to an RNase-free tube and used for further works. RNA isolation from lysed samples followed the manual from the kit Monarch Total RNA Miniprep Kit (New England Biolabs). Elution of RNA was done with 50 μL RNase free water. RNA concentration was measured with a NanoDrop One (Thermo Scientific) at 260 nm. Samples were adjusted to a final RNA concentration of 280 ng in a total volume of 40 μL, dilution was done with RNase-free water. An additional DNase treatment was done by adding 5 μL DNaseI and 5 μL DNase I reaction buffer (New England Biolabs) to the RNA isolates. Digestion was done at 37°C for 10 min and DNase inactivation at 75°C for 10 min. For cDNA synthesis LunaScript RT SuperMix Kit (New England Biolabs) was performed as describe in the manual.

Determination of primer efficiencies was done with diluted cDNA from BG14f_80. cDNA was diluted 1:10, 1:20, 1:40, 1:80, 1:160, 1:320, and 1:640. 1.25 μL of each used for the qRT-PCR reaction. A total volume of 10 μL containing 5 μL Universal qPCR Master Mix (New England Biolabs), 0.25 μL of each oligonucleotide, 1.25 μL sample and 3.25 μL RNase-free water were used. We tested oligonucleotide combinations for the target gene *msfGFP* and housekeeping gene *rpoD* in a CFX Connect Real-Time PCR Detection System (Bio-Rad Laboratories, Hercules, USA) using a protocol described in the manual of Universal qPCR Master Mix (New England Biolabs). CFX Manager software (Bio-Rad Laboratories, Hercules, USA) was used for the calculation of resulting primer efficiencies and an online tool was used to calculated the amplification factor (https://www.thermofisher.com/de/de/home/brands/thermo-scientific/molecular-biology/molecular-biology-learning-center/molecular-biology-resource-library/thermo-scientific-web-tools/qpcr-efficiency-calculator.html). Tested oligonucleotide combinations for *msfGFP* achieved a value of 1.97 and *rpoD* of 2.02 (Udvardi et al., 2008).

Each cDNA sample was diluted 1:10 and analyzed as technical duplicate. Volumes for each reaction are described above. As negative control the same amount of water was added to the reaction instead of cDNA. Examination from resulting Ct values was done with Microsoft Excel and a ΔCt method was applied (Pfaffl, 2001). To exclude genomic DNA in the samples, isolated RNA was used in separate reactions with oligonucleotides for *rpoD*.

### Statistics

Each promoter construct was characterized in 2–3 independent transformations performed on different days. Three clones from each transformant were tested in a Biolector to determine promoter activities, yielding a total of 6–9 biological replicates. For each construct the mean and standard error of the mean was calculated from these combined biological replicates. Significance of difference of the activity of constructs with different spacer lengths was analyzed by one-way ANOVA with Turkey's *post-hoc* comparison. Coefficient of variation, determined by dividing the absolute difference of the predicted and experimental value by the experimental value, was used to compare the accuracy of prediction of stacked promoter activities.

## Results and Discussion

### Identification of the Optimal Distance Between Two Promoters

For the characterization of stacked promoters, we used a mini Tn7 transposon, which integrates as single copy into the *attTn7* site downstream of the *glmS* gene in the genome of *P. putida* KT2440 (Bagdasarian et al., 1981; Choi et al., 2005; Zobel et al., 2015). The transposon is designed to characterize promoters in a reliable and reproducible manner, featuring a BCD2 element to reduce GOI-based expression variability (Mutalik et al., 2013), an *msfGFP* (Landgraf, 2012) gene as reporter, and two flanking terminators to minimize genomic read-through (Figure 1; Zobel et al., 2015).

In order to determine the optimal distance between two promoters, we stacked the 14f and 14g promoters from a previously published synthetic promoter library (Zobel et al., 2015) with spacer sequences with increasing length from 10 to 100 bp by extension at the 3'-end. The promoters are referred to solely by their SEVA code (Zobel et al., 2015; 14a-g, with a being the weakest and g being the strongest) for ease of reference. The spacer was randomly generated (http://www.faculty.ucr.edu/~mmaduro/random.htm) and manually curated for unwanted restriction sites as well as putative ribosome binding sites, −35, and −10 like sequences, which could disturb the analysis due to intrinsic activity. The spacer was created with a GC content of 62%, similar to the genomic average of *P. putida* KT2440 (Nelson et al., 2002).

A promoterless construct BG and wildtype *P. putida* KT2440 were used as negative controls. As positive controls we used the single calibrated promoters described in Zobel et al. (2015), including P_em7_ (Martínez-García et al., 2015) which reaches half of the activity of promoter 14g. With short spacer sequences of <35 bp, the activity of the stacked promoter is lower than that of either of the single promoters (Figure 1). This result is likely caused by steric hindrance of the RNA polymerase holoenzyme, since the combined sigma factor and RNA polymerase cover around 80 bp upstream of the promoter (Schmitz and Galas, 1979). A spacer length of 40–70 and 90–100 bp resulted in activities of comparable strength. A significantly higher activity was observed for 14f_80_14g with an 80 bp spacer compared to all other spacer lengths (one-way ANOVA with Turkey's *post-hoc* comparison).

To exclude that this 80 bp spacer is an outlier due to possible activating sequences, the experiment was repeated with a reverse complement version of the spacer (80i) and a new, independently generated spacer sequence (80new, Supplementary Table 2). All three 80 bp control spacers led to comparable activities, indicating that this distance between two promoters is promoting additive activity of the two promoters. It is interesting to note that the spacer length of 80 bp matches the sequence covered by the RNA polymerase holoenzyme (Schmitz and Galas, 1979), although this correlation should not be confused with causation. Promoterless controls with only 80i and 80new also show no activity, excluding any intrinsic activity from the spacers themselves (Figure 1).

With the 80i spacer a cumulative effect occurred, with the total output of the stacked promoters being higher than the individual activities. However, the output was much lower than the sum of the two individual promoters for each tested spacer length. For further characterization, we used the 80i spacer since it enabled the highest promoter activity.

### Characterization of Context Effects on Stacked Promoters

**Figure 2:**
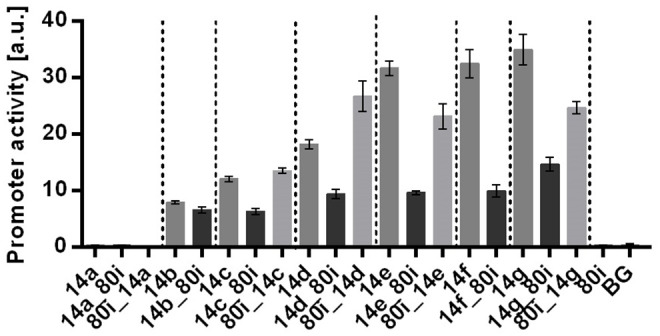
Characterization of context-depended promoter activities using the 80i spacer. Promoters with both up- and downstream spacer were tested. Shown are promoter activities for the original promoters (dark bars) from Zobel et al. (2015) and promoter-spacer (14x_80i) as well as spacer-promoter (80i_14x) combinations (gray bars), where x stands for promoter 14a to 14g. All constructs were genomically integrated in *P. putida* KT2440. Strains were cultured in a BioLector in minimal medium with 20 mM glucose in a 96 well plate Identical strains from at least two different transformations were tested, with three biological replicates each. Vertical dotted lines are separating individual sets. Error bars indicate the standard error of the mean (*n* > 6).

We hypothesized two possible ways how these stacked promoters are affected. The primary hypothesis is that of an effect of the spacer on the promoter. The alternative hypothesis is a mutual influence of one promoter on the other (Callen et al., 2004; Shearwin et al., 2005). To test these hypotheses, we constructed 14 different stacked promoter combinations and 12 controls to determine the influence of the 80i spacer on single promoter activities. Following the rules provided by SEVA (Martínez-García et al., 2015), the promoter is integrated between restriction sites *Pac*I and *Avr*II. The spacer is an additional sequence in the probe vector published by Zobel et al. (2015) and is not replacing any sequences from the original construct. The constructs are named according to their composition, i.e., in 14f_80i promoter 14f is cloned upstream of the 80i spacer. After genomic integration of the Tn7 transposon all strains were characterized in a BioLector system (Figure 2).

The single promoter controls without spacer reached activities that are comparable to those initially described by Zobel et al. (2015). In contrast, single promoter combined with the 80i spacer, either upstream or downstream, were strongly affected in their activity (Figure 2). With downstream placement of the spacer, all promoters were negatively affected, with decreases up to 70% for 14f_80i. In contrast, no clear trend could be discerned with upstream placement of the spacer, with most combinations having decreased activities up to 28%, but 80i_14c gained 12 % and 80i_14d even 50% activity.

These results show that the spacer has a drastic effect on all tested promoters. This is in spite of the fact that the spacer itself doesn't display any promoter activity (Figure 1), nor does it contain any discernible sequences that might affect promoter activity, such as AT-rich UP elements (Estrem et al., 1998). In addition, up- and downstream effects of the spacer are unpredictable. Most of the single promoters show a decreased activity when combined with the spacer, which could be explained by missing upstream activating elements potentially present in the original construct such as the AT-rich *Pac*I restriction site. While no consistent correlation between spacer position and promoter activity is discernible, the results do confirm the primary hypothesis that the activity of the promoters is affected by the spacer.

**Figure 3:**
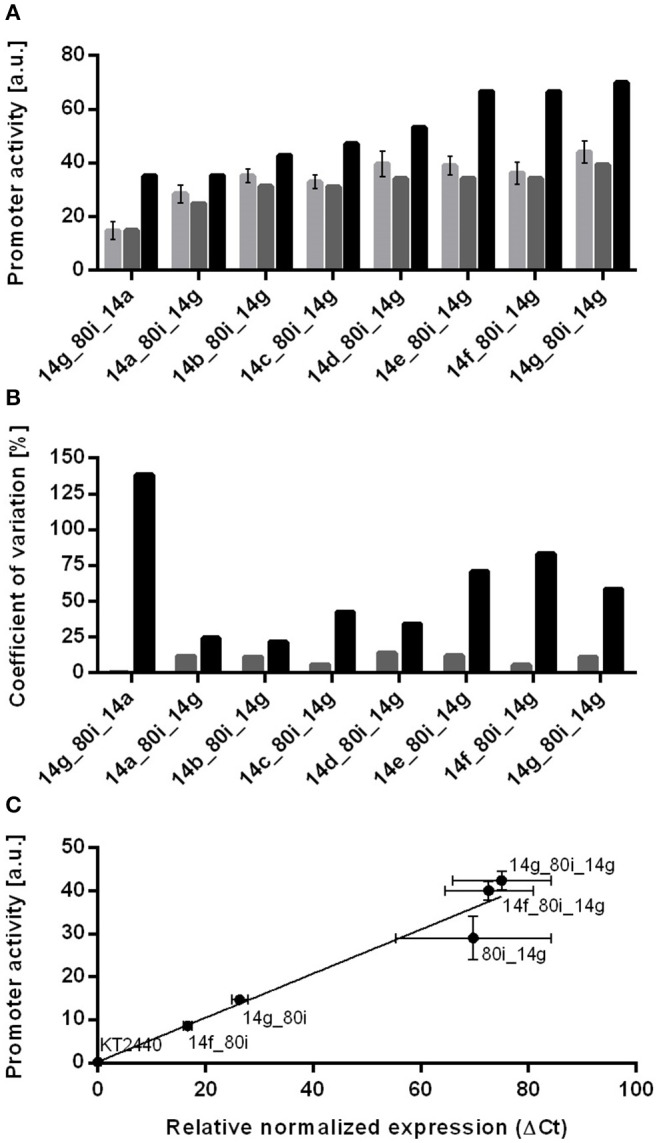
Comparison of experimental values, context-specific and context-unspecific prediction of promoter activities for stacked promoter with the 80i spacer separating promoters 14a to 14g. **(A)** Shown are determined promoter activities for stacked promoters (light gray bars), context-specific activities calculated with context-depended values (gray bars) and context-unspecific activities using original promoter activities (black bars). All constructs were genomically integrated into *P. putida* KT2440. Cultivation was done in a BioLector in minimal medium with 20 mM glucose in a 96 well plate. Identical strains from at least three different transformations were tested, with three biological replicates each. Error bars indicate the standard error of the mean (*n* > 9). **(B)** Coefficient of variation (CV) of context-specific (light gray bars) and context-unspecific (black bars) prediction of resulting promoter activities. **(C)** Plot of *msfGFP* transcription levels normalized to *rpoD* determined by quantitative real time PCR (qRT-PCR) and promoter activities from single promoter controls and stacked promoters. Wild type *P. putida* KT2440 was used as negative control. All constructs are genomically integrated into the genome of *P. putida* KT2440. Cultivations to determine promoter activities were done in a BioLector in minimal medium with 20 mM glucose in a 96 well plate. Cultivation to determine transcription levels was done in 24 well System Duetz plates containing minimal medium with 20 mM glucose. Identical strains from at least two different transformations were tested, with three biological replicates each. Error bars indicate the standard error of the mean (*n* > 6).

To further test if, besides the effect of the spacer, the stacked promoters also affect each other, all seven calibrated promoters were stacked with 14g at the second position. As additional control, the reverse-order combination 14g_80i_14a was also included. As expected from the abovementioned results, all of these combinations led to much lower activities than expected from the sum of the individual promoters disregarding context-effects (Figure 3). Interestingly, the combinations 14a_80i_14g and 14g_80i_14a, which only differ in the order of the promoters, reached completely different activities. During the cloning of these stacks, a triple ‘ffg’ promoter consisting of two 14f and one 14g sequences separated by two spacers was accidentally created, in which the second 14f is shorter by two nucleotides between the −35 and −10 elements. Deriving sequence-function relationships from this promoter would be too complex, but the fact that it is around 45% stronger than the strongest promoter combination 14g_80i_14g makes it useful in applications where very high expression is needed (Lenzen et al., 2019; Bator et al., 2020) (Supplementary Figure 1).

When comparing the activities of these stacks to the single promoter-spacer controls above, it becomes apparent that the immediate context of single promoters is the major determinant for the prediction of promoter activity. The sum of the single promoter activities greatly overestimates the activities of stacked promoters by as much as 140% for the 14g_80i_14a combination (Figure 3). In contrast, the sum of the context-specific controls provides a much more accurate prediction, i.e., 14g_80i + 80i_14a = 14g_80i_14a. In this case, the coefficient of variance between context-specific prediction and experimental values is lower than 15% for all tested combinations. This strongly suggests that, once the direct context of the individual promoters is sufficiently taken into account, the stacked promoters don't affect each other's activity.

Beyond having different promoter contexts, the abovementioned constructs also generate different 5′-terminal mRNA ends, which may cause differences in mRNA stability or translation initiation rates. In order to minimize the effect of these differences, a bicistronic design (Mutalik et al., 2013) was included in the reporter construct. To verify whether the altered expression of context-affected constructs is caused by increased transcription, we performed quantitative real time PCR (qRT-PCR) on selected constructs. Determined transcript levels correlate well with promoter activities derived from fluorescence measurements (*r*^2^ = 0.95, Figure 3), confirming that the spacer influences transcription, rather than translation. Attempts to determine the relative contributions of the first or second promoter by qRT-PCR were inconclusive. In principle, stacked promoters generate two overlapping transcripts of different length, which might be distinguished with different primers pairs. However, longer transcripts show a shift in Ct value compared to shorter amplicons, and suitable primer pairs for similar lengths could not be found (Debode et al., 2017).

### Using an SNP Promoter Library for Stacked Promoters

**Figure 4:**
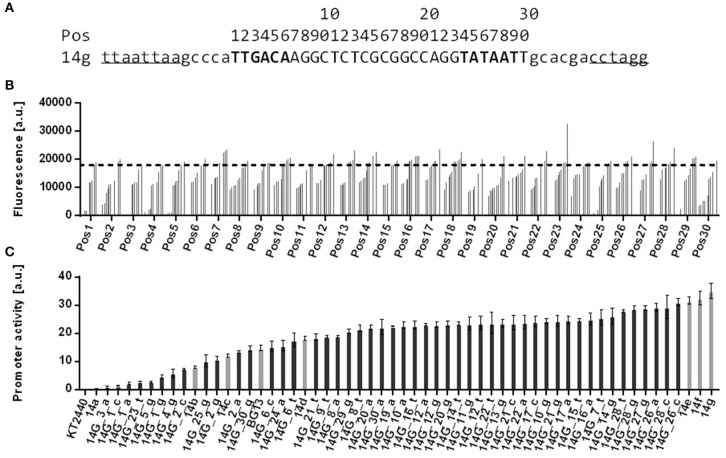
Comparison of screened and characterized promoter sequences based on 14g with single nucleotide polymorphisms. **(A)** Original promoter sequence of 14g with highlighted−35 and−10 elements (in bold). Restriction sites *Pac*I and *Avr*II are underlined and positions of the modified core promoter sequence are given with numbers above the sequence. **(B)** msfGFP fluorescence of *E. coli* PIR2 bearing plasmid pBG14g with degenerate bases at 30 positions along the core promoter sequence. Changed position is shown on the x axis. Determined values are ranked by fluorescence intensity of 14 strains tested for each position. Strains were cultivated in 96 well System Duetz plates with LB medium supplemented with 50 mg L^−1^ kanamycin. Fluorescence and optical density were measured with a plate reader. The dotted line indicates the promoter activity of the 14g control. **(C)** Chosen SNP promoter constructs were genomically integrated into *P. putida* KT2440. Strains are named 14G_##n, whereas ## stands for the position in the promoter sequence and n for a nucleotide (A, C, G or T). Cultivation was done in a BioLector in minimal medium with 20 mM glucose in 96 well plates. Identical strains from at least two different transformations were tested, with three biological replicates each. Error bars indicate the standard error of the mean (*n* > 6).

A change in context greatly affects promoter activitiy, and there are large quantitative differences for each tested promoter-spacer combination. Given that the main variable between these constructs is the promoter sequence, this might be due to specific DNA-DNA interactions between promoter and spacer, which influence promoter activity, or RNA-RNA interactions, which affect RNA stability. To further investigate the sequence-activity relationship, we generated a single nucleotide polymorphism (SNP) library based on promoter 14g (Figure 4). Such a library yields promoters with very similar sequences, but large differences in activity. If the variability of the impact of the spacer on the activity is indeed caused by DNA-DNA or RNA-RNA interactions, using promoters with more similar sequences can be expected to reduce this variability. The library contained 90 different promoter sequences, which were generated in 30 PCR reaction with one degenerate nucleotide in the sequence. Changes of the core promoter sequence were inserted within the −35 element (position 1–6), in the interspaced region (position 7–23, 30), and in the −10 element (position 24–29). For each position four different promoters can occur, of which one will correspond to the original 14g sequence. Initial screening of mini libraries (14 clones each) of these low degeneracy promoters was performed in plasmid-bearing *E. coli* PIR2 strains by analyzing msf GFP fluorescence. Aberrations compared to original pBG14g-bearing *E. coli* PIR2 strains were recognized (Figure 4). For nearly each position clones were found with either a higher or lower fluorescence signal than the original pBG14g plasmid. Variations in the interspaced region generally had a lower effect on expression strength, while changing single nucleotides in the −35 and −10 consensus sequences yielded more clones with decreased promoter activity, as expected (Lodge et al., 1990; McLean et al., 1997). We therefore focused further characterization on these elements in order to obtain a set of promoters with a range of activity that is comparable to the previously described calibrated promoter library from Zobel et al. (2015) (Figure 4).

**Figure 5:**
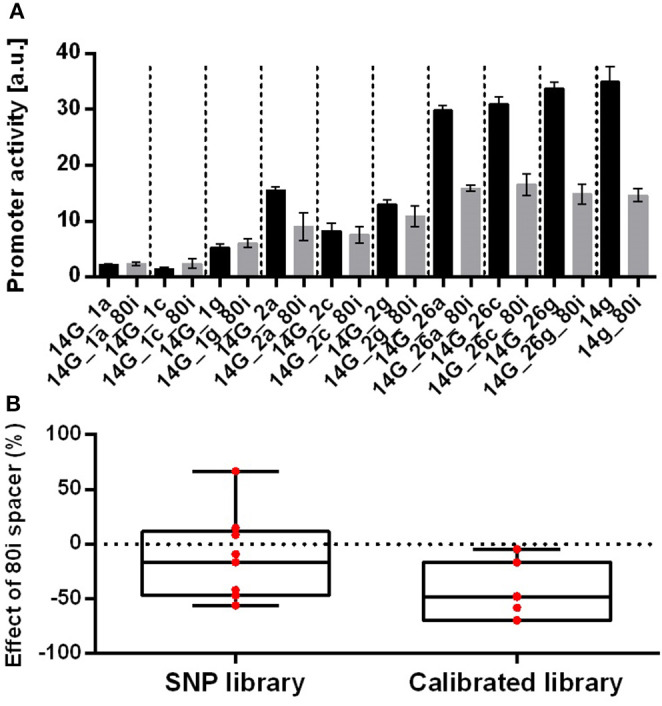
Characterization of the effect of the 80i spacer on a single nucleotide exchange promoter library based on promoter 14g genomically integrated in *P. putida* KT2440. **(A)** Promoter activities derived from GFP fluorescence analysis of SNP promoters with (gray bars) and without (black bars) downstream 80i spacer. Cultivation was done in a BioLector in minimal medium with 20 mM glucose in 96 well plates. Identical strains from at least two different transformations were tested, with three biological replicates each. Vertical dotted lines are separating individual sets. Error bars indicate the standard error of the mean (*n* > 6). **(B)** Box and whiskers plot of the relative effect of the downstream 80i spacer on promoter activities of the SNP library and the original calibrated promoter library from Zobel et al. (2015). The effect of the spacer is calculated as the % change of the promoter with downstream 80i sequence compared to the original promoter. Data points are indicated in red.

After initial screening of positions in the SNP promoter sequences, we selected three such positions within the −35 or −10 elements for further characterization. Introducing a degenerate base in these three positions yields nine different promoters with a good spread of activity, and these promoters were combined with the 80i spacer in the downstream position (Figure 5). Changing position 26 in the −10 sequence resulted in a slightly decreased activity, while changes in positions 1 and 2 in the −35 sequence yielded larger decreases, which is in accordance with Lodge et al. (1990).

We have seen that small changes in the 14g promoter sequence can strategically affect activity in a mini-promoter-library (Supplementary Table 10). In spite of the relative uniformity of the promoter sequences in this library, combination of these promoters with the 80i spacer again strongly affected the activities with both in- and decreases up to 66 %. The reduced sequence variability did not reduce the quantitative variability of the spacer effect compared to the CalPro library from Zobel et al. (2015). Both have a high coefficient of variation of 40% for the SNP library and 25% for the CalPro library (Figure 5). This strongly suggests that the promoter sequence *per se* does not cause the context-dependent effect, suggesting that other factors such as the varying transcription-initiation rates are in play.

## Conclusion

In this work, we aimed to increase the composability and predictability of synthetic promoters by investigating the effects of differing contexts on their activity. In the combination of two promoters, the length of the spacer region is crucial for reaching higher and cumulative effects, with 80 bp being the optimal. Even though the spacer sequence has no intrinsic activity, it strongly and unpredictably influences the activity of promoters by up- and downstream effects. By accounting for this influence, the activity of two stacked promoters can be accurately predicted with coefficients of variance below 15%. A strong reduction of sequence variability was achieved using an SNP library, but this reduction did not reduce the quantitative variability of the spacer effect. This strongly indicates that nucleotide-nucleotide interactions between promoter and spacer do not play a prominent role. Clearly, context-specific effects of synthetic promoters are not yet fully understood in *Pseudomonas*. Although the semi-empirical approach for prediction of stacked promoter activities provides an accurate workaround to this, further work is needed to understand the fundamental interaction of genetic elements and their surroundings.

## Data Availability Statement

The datasets generated for this study are available on request to the corresponding author.

## Author Contributions

NW and SK designed the experiments. SK performed all molecular engineering and experiments, prepared figures and wrote the manuscript. NW supervised the study and edited the manuscript. LB advised on all experiments, analyzed and discussed data, and edited the manuscript.

## Conflict of Interest

The authors declare that the research was conducted in the absence of any commercial or financial relationships that could be construed as a potential conflict of interest.
