# Supplementary material for: Characterization of Context-Dependent Effects on Synthetic Promoters
Source: Front Bioeng Biotechnol. 2020 Jun 12;8:551. doi: 10.3389/fbioe.2020.00551 (PMC7303508; doi:10.3389/fbioe.2020.00551)
Supplement: Supplementary file 1 [file Data_Sheet_1.docx]

***Supplementary Material***

**Characterization of context-dependent effects on synthetic promoters**

**Sebastian Köbbing^1^, Lars M. Blank^1^ and Nick Wierckx^1,2*^**

^1^ Institute of Applied Microbiology - iAMB, Aachen Biology and Biotechnology – ABBt, RWTH Aachen University, Aachen, Germany

^2^ Institute of Bio- and Geosciences (IBG-1: Biotechnology), Forschungszentrum Jülich GmbH, Jülich, Germany

***Correspondence**:

Nick Wierckx

n.wierckx@fz-juelich.de

# Support

## Supplementary Tables

Oligonucleotides used in this study are listed in Supplementary Table 1. Spacer sequences are given in Supplementary Table 2. Construction of different length spacer sequences up to 40 bp is shown in Supplementary Table 3, Supplementary Table 4 points out combinations for 50 to 100 bp. Supplementary Table 5 is listing all oligonucleotide combinations to generate stacked promoters. Supplementary Table 6 shows which oligonucleotides were used to create controls for each promoter and position. Supplementary Table 7 shows how spacer alone controls were amplified. PCR program used for the generation of stacked promoters given in Supplementary Table 8. Strains and plasmid generated and used in this study are listed in Supplementary Table 9. Supplementary Table 10 shows promoter sequences of the SNP library based on promoter 14g with corresponding activities compared to the original sequence.

Supplementary Table 1: Oligonucleotides used in this study. Promoter sequences and restriction sites are indicated by different colors or are underlined.

| Name | Sequence 5´-3´^a^ | Reference |
| --- | --- | --- |
| SK4 | AGTCAGAGTTACGGAATTGTAGG | Zobel *et al*.(Zobel et al., 2015) |
| SK5 | GTCGAGAAAATTGCCGAGCT | Zobel *et al*.(Zobel et al., 2015) |
| SK2 | ACACCATAGGTCAGGGTAGTC | This work |
| SK43 | ATCAAACATCGACCCACGGCGTAAC | This work |
| SK11 | CGCTTAATTAAGCCCGTTGACATGACATGGTTTTGAGGGTATAATGTGGCGACCTTAATTAAGCCCATTGACAAG | This work |
| SK34 | CGCTTAATTAAGCCCGTTGACATGACATGGTTTTGAGGGTATAATGTGGCGAGGACGAGTCACCATGTGCCAGCCCATTGACAAGGCTCTCG | This work |
| SK36 | CGCTTAATTAAGCCCGTTGACATGACATGGTTTTGAGGGTATAATGTGGCGAGGACGAGTCACCATGTGCCAGGGGCGATAAGCCCATTGACAAGGCTCTCG | This work |
| SK38 | CGCTTAATTAAGCCCGTTGACATGACATGGTTTTGAGGGTATAATGTGGCGAGGACGAGTCACCATGTGCCAGGGGCGATAACGATCGGTGGGCCCATTGACAAGGCTCTCG | This work |
| SK50 | GCGTTAATTAAGCCCGTTGACATGACATGGTTTTGAGGGTAT5AATGTGGCGAGGACGAGTCACCATGTGCCAGGGGCGATAACGATCGGTGGGAGTATTCAT | This work |
| SK51 | GCGCCTAGGTCGTGCAATTATACCTGGCCGCGAGAGCCTTGTCAATGGGCATGAATACTCCCACCGATCG | This work |
| SK52 | GCGCCTAGGTCGTGCAATTATACCTGGCCGCGAGAGCCTTGTCAATGGGCCTTCACCGCGATGAATACTCCCACCGATCG | This work |
| SK53 | GCGCCTAGGTCGTGCAATTATACCTGGCCGCGAGAGCCTTGTCAATGGGCAACCCAGCGCCTTCACCGCGATGAATACTCCCACCGATCG | This work |
| SK54 | GCGCCTAGGTCGTGCAATTATACCTGGCCGCGAGAGCCTTGTCAATGGGCTTCCCACGCGAACCCAGCGCCTTCACCGCGATGAATACTCCCACCGATCG | This work |
| SK55 | GCGCCTAGGTCGTGCAATTATACCTGGCCGCGAGAGCCTTGTCAATGGGCACAAGCACCTTTCCCACGCGAACCCAGCGCCTTCACCGCGATGAATACTCCCACCGATCG | This work |
| SK56 | GCGCCTAGGTCGTGCAATTATACCTGGCCGCGAGAGCCTTGTCAATGGGCCTGCTGGGACACAAGCACCTTTCCCACGCGAACCCAGCGCCTTCACCGCGATGAATACTCCCACCGATCG | This work |
| SK57 | GCGTTAATTAAGCCCGTTG | This work |
| SK58 | GCGCCTAGGTCGTGCAATTATAC | This work |
| SK93 | GCGTTAATTAAGCCCGTTGACATGACATGGTTTTGAGGGTATAATGTGGCGATTCCCACGCGAACCCAGCGCCTTCACCGCGATGAATACTCCCACCGATCG | This work |
| SK94 | GCGCCTAGGTCGTGCAATTATACCTGGCCGCGAGAGCCTTGTCAATGGGCGGACGAGTCACCATGTGCCAGGGGCGATAACGATCGGTGGGAGTATTCAT | This work |
| SK97 | GCGTTAATTAACTAGGTTGACATGGATATAATGTATGTATTCCCACGCGAACCCAGCGCCTTCACCGCGATGAATACTCCCACCGATCG | This work |
| SK103 | GCGTTAATTAACTAGGTTGAC | This work |
| SK98 | GCGTTAATTAATTTATTTGACATGCGTGATGTTTAGAATTATAATTTGGGGATTCCCACGCGAACCCAGCGCCTTCACCGCGATGAATACTCCCACCGATCG | This work |
| SK104 | GCGTTAATTAATTTATTTGACATG | This work |
| SK99 | GCGTTAATTAAGTGAATTGACATGTCAATTTTTATGTTGTATAATATAACTATTCCCACGCGAACCCAGCGCCTTCACCGCGATGAATACTCCCACCGATCG | This work |
| SK105 | GCGTTAATTAAGTGAATTGACAT | This work |
| SK100 | GCGTTAATTAATCTACTTGACATCCGACATTCGCGACTGTATAATAAGTTGATTCCCACGCGAACCCAGCGCCTTCACCGCGATGAATACTCCCACCGATCG | This work |
| SK106 | GCGTTAATTAATCTACTTGACAT | This work |
| SK101 | GCGTTAATTAACGGAGTTGACAACACTCGAAAAGCCGAGTATAATCAGATGATTCCCACGCGAACCCAGCGCCTTCACCGCGATGAATACTCCCACCGATCG | This work |
| SK107 | GCGTTAATTAACGGAGTTGAC | This work |
| SK102 | GCGTTAATTAAGCCCATTGACAAGGCTCTCGCGGCCAGGTATAATTGCACGATTCCCACGCGAACCCAGCGCCTTCACCGCGATGAATACTCCCACCGATCG | This work |
| SK108 | GCGTTAATTAAGCCCATTGACA | This work |
| SK122 | GCGCCTAGGTAGTTATATTATACAACATAAAAATTGACATGTCAATTCACGGACGAGTCACCATGTGCCAGGGGCGATAACGATCGGTGGGAGTATTCAT | This work |
| SK124 | GCGCCTAGGTAGTTATATTA | This work |
| SK123 | GCGCCTAGGTACATACATTATATCCATGTCAACCTAGGGACGAGTCACCATGTGCCAGGGGCGATAACGATCGGTGGGAGTATTCAT | This work |
| SK125 | GCGCCTAGGTACATACATTATATC | This work |
| SK135 | GCGCCTAGGTCATCTGATTATACTCGGCTTTTCGAGTGTTGTCAACTCCGGGACGAGTCACCATGTGCCAGGGGCGATAACGATCGGTGGGAGTATTCAT | This work |
| SK137 | GCGCCTAGGTCATCTG | This work |
| SK136_ | GCGCCTAGGTCAACTTATTATACAGTCGCGAATGTCGGATGTCAAGTAGAGGACGAGTCACCATGTGCCAGGGGCGATAACGATCGGTGGGAGTATTCAT | This work |
| SK138 | GCGCCTAGGTCAACTTA | This work |
| SK272 | GCGTTAATTAAGCCCGTTGACATGACATGGTTTTGAGGGTATAATGTGGCGAAACTGCCTTGCTTCTCGGTGTGATACCCCCGTCAGCCGCCCGCGGTGGTG | This work |
| SK273 | GCGCCTAGGTCGTGCAATTATACCTGGCCGCGAGAGCCTTGTCAATGGGCGTGCCCAATACTCCAATCGGCTTTCACGTGCACCACCGCGGGCGGCTGAC | This work |
| SK274 | CGCTTAATTAAAACTGCCTTGCTTCTCGGTG | This work |
| SK275 | CGCCCTAGGGTGCCCAATACTCCAATCGG | This work |
| SK145 | CGCTTAATTAATTCCCACGCGAACCC | This work |
| SK146 | CGCCCTAGGGGACGAGTCACCATGTG | This work |
| SK334 | AGGCATTCGTGAAGTCATGG | This work |
| SK335 | ATGTAACCGCTGAGAACGTC | This work |
| SK337 | CACCGCAGACAAACAGAAGA | This work |
| SK336 | ACTGGGTGGACAGGTAGTGG | This work |
| SK63 | CGCTTAATTAAGCCCA**N**TGACAAGGC | This work |
| SK64 | CGCTTAATTAAGCCCAT**N**GACAAGGCT | This work |
| SK65 | CGCTTAATTAAGCCCATT**N**ACAAGGCTC | This work |
| SK66 | CGCTTAATTAAGCCCATTG**N**CAAGGCTCT | This work |
| SK67 | CGCTTAATTAAGCCCATTGA**N**AAGGCTCTC | This work |
| SK68 | CGCTTAATTAAGCCCATTGAC**N**AGGCTCTCG | This work |
| SK69 | CGCTTAATTAAGCCCATTGACA**N**GGCTCTCGC | This work |
| SK70 | CGCTTAATTAAGCCCATTGACAA**N**GCTCTCGCG | This work |
| SK71 | CGCTTAATTAAGCCCATTGACAAG**N**CTCTCGCGG | This work |
| SK72 | CGCTTAATTAAGCCCATTGACAAGG**N**TCTCGCGGC | This work |
| SK73 | CGCTTAATTAAGCCCATTGACAAGGC**N**CTCGCGGCC | This work |
| SK74 | CGCTTAATTAAGCCCATTGACAAGGCT**N**TCGCGGCCA | This work |
| SK75 | CGCTTAATTAAGCCCATTGACAAGGCTC**N**CGCGGCCAG | This work |
| SK76 | CGCTTAATTAAGCCCATTGACAAGGCTCT**N**GCGGCCAGG | This work |
| SK77 | CGCTTAATTAAGCCCATTGACAAGGCTCTC**N**CGGCCAGGT | This work |
| SK78 | CGCTTAATTAAGCCCATTGACAAGGCTCTCG**N**GGCCAGGTA | This work |
| SK79 | CGCTTAATTAAGCCCATTGACAAGGCTCTCGC**N**GCCAGGTAT | This work |
| SK80 | CGCTTAATTAAGCCCATTGACAAGGCTCTCGCG**N**CCAGGTATA | This work |
| SK81 | CGCTTAATTAAGCCCATTGACAAGGCTCTCGCGG**N**CAGGTATAA | This work |
| SK82 | CGCTTAATTAAGCCCATTGACAAGGCTCTCGCGGC**N**AGGTATAAT | This work |
| SK83 | CGCTTAATTAAGCCCATTGACAAGGCTCTCGCGGCC**N**GGTATAATT | This work |
| SK84 | CGCTTAATTAAGCCCATTGACAAGGCTCTCGCGGCCA**N**GTATAATTG | This work |
| SK85 | CGCTTAATTAAGCCCATTGACAAGGCTCTCGCGGCCAG**N**TATAATTGC | This work |
| SK86 | CGCTTAATTAAGCCCATTGACAAGGCTCTCGCGGCCAGG**N**ATAATTGCA | This work |
| SK87 | CGCTTAATTAAGCCCATTGACAAGGCTCTCGCGGCCAGGT**N**TAATTGCAC | This work |
| SK88 | CGCTTAATTAAGCCCATTGACAAGGCTCTCGCGGCCAGGTA**N**AATTGCACG | This work |
| SK89 | CGCTTAATTAAGCCCATTGACAAGGCTCTCGCGGCCAGGTAT**N**ATTGCACGA | This work |
| SK90 | CGCTTAATTAAGCCCATTGACAAGGCTCTCGCGGCCAGGTATA**N**TTGCACGAC | This work |
| SK91 | CGCTTAATTAAGCCCATTGACAAGGCTCTCGCGGCCAGGTATAA**N**TGCACGACC | This work |
| SK92 | CGCTTAATTAAGCCCATTGACAAGGCTCTCGCGGCCAGGTATAAT**N**GCACGACCT | This work |

^a^ Restriction sites *Pac*I and *Avr*II are underlined. Depending on the included promoter, oligonucleotides are colored differently: yellow letters presenting promoter 14a, orange 14b, purple 14c, green 14d, grey 14e, red 14f, and blue 14g. Furthermore, highlighted bolt typed letters are indicating random nucleotides. N stands for nucleotides a, c, g, or t.

Supplementary Table 2: Spacer sequences characterized in this study.

| Distance [bp] | Sequence |
| --- | --- |
| 10 | CCATAATTAA |
| 20 | GGACGAGTCACCATGTGCCA |
| 30 | GGACGAGTCACCATGTGCCAGGGGCGATAA |
| 40 | GGACGAGTCACCATGTGCCAGGGGCGATAACGATCGGTGG |
| 50 | GGACGAGTCACCATGTGCCAGGGGCGATAACGATCGGTGGGAGTATTCAT |
| 60 | GGACGAGTCACCATGTGCCAGGGGCGATAACGATCGGTGGGAGTATTCATCGCGGTGAAG |
| 70 | GGACGAGTCACCATGTGCCAGGGGCGATAACGATCGGTGGGAGTATTCATCGCGGTGAAGGCGCTGGGTT |
| 80 | GGACGAGTCACCATGTGCCAGGGGCGATAACGATCGGTGGGAGTATTCATCGCGGTGAAGGCGCTGGGTTCGCGTGGGAA |
| 80i | TTCCCACGCGAACCCAGCGCCTTCACCGCGATGAATACTCCCACCGATCGTTATCGCCCCTGGCACATGGTGACTCGTCC |
| 80new | AACTGCCTTGCTTCTCGGTGTGATACCCCCGTCAGCCGCCCGCGGTGGTGCACGTGAAAGCCGATTGGAGTATTGGGCAC |
| 90 | GGACGAGTCACCATGTGCCAGGGGCGATAACGATCGGTGGGAGTATTCATCGCGGTGAAGGCGCTGGGTTCGCGTGGGAAAGGTGCTTGT |
| 100 | GGACGAGTCACCATGTGCCAGGGGCGATAACGATCGGTGGGAGTATTCATCGCGGTGAAGGCGCTGGGTTCGCGTGGGAAAGGTGCTTGTGTCCCAGCAG |

Supplementary Table 3: Oligonucleotide combinations used for the generation of spacer sequences with a length up to 40 bp.

| Distance [bp] | Promoter | Forward oligonucleotide | Reverse oligonucleotide | template |
| --- | --- | --- | --- | --- |
| 10 | 14f_10_14g | SK11 | SK2 | pBG14g |
| 20 | 14f_20_14g | SK34 | SK2 | pBG14g |
| 30 | 14f_30_14g | SK36 | SK2 | pBG14g |
| 40 | 14f_40_14g | SK38 | SK2 | pBG14g |

Supplementary Table 4: Oligonucleotide combinations used for the generation of spacer sequences with a spacer length from 50 to100 bp. PCR was used to generate dsDNA fragment from two long oligonucleotides containing restrictions sites and promoter sequences. Block forming is achieved by complementary 3´-ends.

| Distance [bp] | Promoter | 1^st^ PCR  Forward oligonucleotide 1^st^ promoter | Reverse oligonucleotide 2^nd^ promoter | 2^nd^ PCR  Forward oligonucleotide 1^st^ promoter | Reverse oligonucleotide 2^nd^ promoter |
| --- | --- | --- | --- | --- | --- |
| 50 | 14f_50_14g | SK50 | SK51 | SK57 | SK58 |
| 60 | 14f_60_14g | SK50 | SK52 | SK57 | SK58 |
| 70 | 14f_70_14g | SK50 | SK53 | SK57 | SK58 |
| 80 | 14f_80_14g | SK50 | SK54 | SK57 | SK58 |
| 90 | 14f_90_14g | SK50 | SK55 | SK57 | SK58 |
| 100 | 14f_100_14g | SK50 | SK56 | SK57 | SK58 |
| 80i | 14f_80i_14g | SK93 | SK94 | SK57 | SK58 |
| 80new | 14f_80new_14g | SK272 | SK273 | SK57 | SK58 |

Supplementary Table 5: Oligonucleotide combinations used for the generation of stacked promoters with a spacer distance of 80 bp. PCR was used to generate dsDNA fragments from two long oligonucleotides containing restrictions sites and promoter sequences. Block forming is achieved by complementary 3´-ends.

| Promoter combination | 1^st^ PCR  Forward oligonucleotide  1^st^ promoter | Reverse oligonucleotide  2^nd^ promoter | 2^nd^ PCR  Forward oligonucleotide  1^st^ promoter | Reverse oligonucleotide  2^nd^ promoter |
| --- | --- | --- | --- | --- |
| 14a_80i_14g | SK97 | SK94 | SK103 | SK58 |
| 14b_80i_14g | SK98 | SK94 | SK104 | SK58 |
| 14c_80i_14g | SK99 | SK94 | SK105 | SK58 |
| 14d_80i_14g | SK100 | SK94 | SK106 | SK58 |
| 14e_80i_14g | SK101 | SK94 | SK107 | SK58 |
| 14f_80i_14g | SK50 | SK94 | SK57 | SK58 |
| 14g_80i_14g | SK102 | SK94 | SK108 | SK58 |
| 14g_840i_14a | SK102 | SK123 | SK108 | SK125 |
| 14b_80i_14c | SK98 | SK122 | SK104 | SK124 |
| 14e_80i_14d | SK101 | SK107 | SK136 | SK138 |
| 14d_80i_14e | SK100 | SK106 | SK135 | SK137 |

Supplementary Table 6: Oligonucleotides used for generation of promoter position controls for two positions and different promoters. Constructed stacking promoter containing vectors was used as a template.

| 1^st^ and 2^nd^ position promoter control | Forward oligonucleotide | Reverse oligonucleotide | Template |
| --- | --- | --- | --- |
| 14a_80i | SK43 | SK146 | pBG14a/14g |
| 14b_80i | SK43 | SK146 | pBG14b/14c |
| 14c_80i | SK43 | SK146 | pBG14c/14e |
| 14d_80i | SK43 | SK146 | pBG14e/14d |
| 14e_80i | SK43 | SK146 | pBG14d/14e |
| 14f_80i | SK43 | SK146 | pBG14f/14g |
| 14g_80i | SK43 | SK146 | pBG14g/14g |
| 14g_1x^a^_80i | SK63 | SK146 | pBG14g_80 |
| 14g_2x^a^_80i | SK64 | SK146 | pBG14g_80 |
| 14g_26x^a^_80i | SK88 | SK146 | pBG14g_80 |
| 14f_80new | SK43 | SK275 | pBG14f/80new/14g |
| 80_14a | SK145 | SK2 | pBG14g/14a |
| 80i_14c | SK145 | SK2 | pBG14b/14c |
| 80i_14d | SK145 | SK2 | pBG14e/14d |
| 80i_14e | SK145 | SK2 | pBG14d/14e |
| 80i_14g | SK145 | SK2 | pBG14g/14g |
| 80new_14g | SK274 | SK2 | pBG14f/80new/14g |

a) x stands for nucleotide A, C, G or T

Supplementary Table 7: Oligonucleotide combinations used for the generation of spacer control without promoters. As a template previously, generated vectors with a distinct spacer sequence were used.

| Distance [bp] control | Forward oligonucleotide | Reverse oligonucleotide | Template |
| --- | --- | --- | --- |
| pBG_80i | SK145 | SK146 | pBG14f_80i_14g |
| pBG_80new | SK274 | SK275 | pBG14f_80new_14g |

Supplementary Table 8: Composition of used PCR reaction mix for the generation of stacked promoters and executed PCR program consisting of two PCR processes.

| PCR reaction | Component | Volume |  |
| --- | --- | --- | --- |
|  | Q5 polymerase | 0.5 µL | |
|  | Q5 buffer | 10 µL | |
|  | dNTPs | 5 µL | |
|  | 1^st^ oligonucleotides | 0.3 µL each (0.01 µmol) | |
|  | Water | To 45 µL | |
| First PCR | **Temperature** | **Time** | **Number of cycles** |
| Initial denaturation | 98°C | 2 min | 1 |
| Denaturation | 95°C | 20 sec | 1 |
| Annealing | 95-58°C | 1 min,  0.5°C/sec | 1 |
| Extension | 72 | 1 min | 1 |
| Final extension | 72 | 5 min | 1 |
| Second PCR | **Temperature** | **Time** | **Number of cycles** |
| Addition of secondary oligonucleotides, each 2.5 µL (0.005 µmol) | | | |
| Initial Denaturation | 98°C | 2 min | 1 |
| Denaturation | 95 | 20 sec | 30 |
| Annealing | 61 | 20 sec | 30 |
| Extension | 72 | 30 sec | 30 |
| Final extension | 72 | 5 min | 1 |

Supplementary Table 9: Complete list of used and generated strains and plasmids in this work.

| Strain | Description | Reference |
| --- | --- | --- |
| *E. coli* |  |  |
| HB101 | *F^−^ mcrB mrr hsdS20*(*rB^−^ mB^−^*) *recA13 leuB6 ara-14 proA2 lacY1 galK2 xyl-5 mtl-1 rpsL20*(Sm^R^) *gln V44λ^−^* | Boyer and Roulland-Dussoix (1969) |
| CC118λpir | Δ(*ara*-*leu*) *araD* Δ*lacX74 galE galK phoA20 thi-1 rpsE rpoB argE*(*Am*) *recA1,* lysogenized with λpir phage | Herrero et al. (1990) |
| PIR2 | F^‑^ Δ*lac169 rpoS* (Am) *robA1 creC510 hsdR514 endA reacA1 uidA* (Δ*Mlui*)::*pir* | Life Technologies |
| *E. coli* DH5αλ pir | *endA1 hsdR17 glnV44* (= *supE44*) *thi-1 recA1 gyrA96 relA1* φ*80dlac*Δ(*lacZ*)*M15* Δ(*lacZYA*-*argF*)*U169 zdg*-*232*::Tn10 *uidA*::*pir*+ | de Lorenzo lab |
| *P. putida* |  |  |
| KT2440 | Wild-type strain derived of *P. putida* mt-2 cured of the pWW0 plasmid | Bagdasarian et al. (1981) |
| BG | Gm^R^, *P. putida* KT2440 with genomic insertion of pBG | Zobel et al. (2015) |
| BG13 | Gm^R^, *P. putida* KT2440 with genomic insertion of pBG13 | Zobel et al. (2015) |
| BG14a | Gm^R^, *P. putida* KT2440 with genomic insertion of pBG14a | Zobel et al. (2015) |
| BG14b | Gm^R^, *P. putida* KT2440 with genomic insertion of pBG14b | Zobel et al. (2015) |
| BG14c | Gm^R^, *P. putida* KT2440 with genomic insertion of pBG14c | Zobel et al. (2015) |
| BG14d | Gm^R^, *P. putida* KT2440 with genomic insertion of pBG14d | Zobel et al. (2015) |
| BG14e | Gm^R^, *P. putida* KT2440 with genomic insertion of pBG14e | Zobel et al. (2015) |
| BG14f | Gm^R^, *P. putida* KT2440 with genomic insertion of pBG14f | Zobel et al. (2015) |
| BG14g | Gm^R^, *P. putida* KT2440 with genomic insertion of pBG14g | Zobel et al. (2015) |
| BG14f_##_14g | Gm^R^, *P. putida* KT2440 with genomic insertion of pBG14f_##_14g, spacer with varying length from ten to 100 bp | This work |
| BG_80i | Gm^R^, *P. putida* KT2440 with genomic insertion of pBG_80i | This work |
| BG_80new | Gm^R^, *P. putida* KT2440 with genomic insertion of pBG_80new | This work |
| BG14f_80i_14g | Gm^R^, *P. putida* KT2440 with genomic insertion of pBG14f_80i_14g | This work |
| BG14a_80i_14g | Gm^R^, *P. putida* KT2440 with genomic insertion of pBG14a_80i_14g | This work |
| BG14b_80i_14g | Gm^R^, *P. putida* KT2440 with genomic insertion of pBG14b_80i_14g | This work |
| BG14c_80i_14g | Gm^R^, *P. putida* KT2440 with genomic insertion of pBG14c_80i_14g | This work |
| BG14d_80i_14g | Gm^R^, *P. putida* KT2440 with genomic insertion of pBG14d_80i_14g | This work |
| BG14e_80i_14g | Gm^R^, *P. putida* KT2440 with genomic insertion of pBG14e_80i_14g | This work |
| BG14f_80i_14g | Gm^R^, *P. putida* KT2440 with genomic insertion of pBG14f_80i_14g | This work |
| BG14g_80i_14g | Gm^R^, *P. putida* KT2440 with genomic insertion of pBG14g_80i_14g | This work |
| BG14f_80i_14f_80i_14g | Gm^R^, *P. putida* KT2440 with genomic insertion of pBG14f_80i_14f_80i_14g | This work |
| BG14g_80i_14a | Gm^R^, *P. putida* KT2440 with genomic insertion of pBG14g_80i_14a | This work |
| BG14a_80i | Gm^R^, *P. putida* KT2440 with genomic insertion of pBG14a_80i | This work |
| BG14b_80i | Gm^R^, *P. putida* KT2440 with genomic insertion of pBG14b_80i | This work |
| BG14c_80i | Gm^R^, *P. putida* KT2440 with genomic insertion of pBG14c_80i | This work |
| BG14d_80i | Gm^R^, *P. putida* KT2440 with genomic insertion of pBG14d_80i | This work |
| BG14e_80i | Gm^R^, *P. putida* KT2440 with genomic insertion of pBG14e_80i | This work |
| BG14f_80i | Gm^R^, *P. putida* KT2440 with genomic insertion of pBG14f_80i | This work |
| BG14g_80i | Gm^R^, *P. putida* KT2440 with genomic insertion of pBG14g_80i | This work |
| BG_80i_14a | Gm^R^, *P. putida* KT2440 with genomic insertion of pBG_80i_14a | This work |
| BG_80i_14c | Gm^R^, *P. putida* KT2440 with genomic insertion of pBG_80i_14c | This work |
| BG_80i_14d | Gm^R^, *P. putida* KT2440 with genomic insertion of pBG_80i_14d | This work |
| BG_80i_14e | Gm^R^, *P. putida* KT2440 with genomic insertion of pBG_80i_14e | This work |
| BG_80i_14g | Gm^R^, *P. putida* KT2440 with genomic insertion of pBG_80i_14g | This work |
| BG14f_80new | Gm^R^, *P. putida* KT2440 with genomic insertion of pBG14f_80new | This work |
| BG_80new_14g | Gm^R^, *P. putida* KT2440 with genomic insertion of pBG_80new_14f | This work |
| BG14f_80new_14g | Gm^R^, *P. putida* KT2440 with genomic insertion of pBG14f_80new_14g | This work |
| BG14g_SPL_PosZZ_N | Gm^R^, *P. putida* KT2440 with genomic insertion of pBG14g_SPL_PosZZ_N | This work |
| BG14g_1a_80i | Gm^R^, *P. putida* KT2440 with genomic insertion of pBG14f_1a_80i | This work |
| BG14g_1c_80i | Gm^R^, *P. putida* KT2440 with genomic insertion of pBG14g_1c_80i | This work |
| BG14g_1g_80i | Gm^R^, *P. putida* KT2440 with genomic insertion of pBG14g_1g_80i | This work |
| BG14g_1t_80i | Gm^R^, *P. putida* KT2440 with genomic insertion of pBG14g_1t_80i | This work |
| BG14g_2a_80i | Gm^R^, *P. putida* KT2440 with genomic insertion of pBG14g_2a_80i | This work |
| BG14g_2c_80i | Gm^R^, *P. putida* KT2440 with genomic insertion of pBG14g_2c_80i | This work |
| BG14g_2g_80i | Gm^R^, *P. putida* KT2440 with genomic insertion of pBG14g_2g_80i | This work |
| BG14g_2t_80i | Gm^R^, *P. putida* KT2440 with genomic insertion of pBG14g_2t_80i | This work |
| BG14g_26a_80i | Gm^R^, *P. putida* KT2440 with genomic insertion of pBG14g_26a_80i | This work |
| BG14g_26c_80i | Gm^R^, *P. putida* KT2440 with genomic insertion of pBG14g_26c_80i | This work |
| BG14g_26g_80 | Gm^R^, *P. putida* KT2440 with genomic insertion of pBG14g_26g_80i | This work |
| BG14g_26t_80i | Gm^R^, *P. putida* KT2440 with genomic insertion of pBG14g_26t_80i | This work |
| Plasmids |  |  |
| pRK600 | Cm^R^, oriColE1, *tra* + *mob* + of RK2 | Keen et al. (1988) |
| pTnS-1 | Ap^R^, oriR6K, *TnSABC*+*D* operon | Choi et al. (2005) |
| pBG | Km^R^, Gm^R^,oriR6K, Tn7L and Tn7R extremes, BCD2−*msfgfp* fusion | Zobel et al. (2015) |
| pBG13 | Km^R^, Gm^R^, oriR6K, pBG-derived, promoter P_em7_ | Martínez-García et al. (2015) |
| pBG14a | Km^R^, Gm^R^, oriR6K, pBG-derived, promoter 14a | Zobel et al. (2015) |
| pBG14b | Km^R^, Gm^R^, oriR6K, pBG-derived, promoter 14b | Zobel et al. (2015) |
| pBG14c | Km^R^, Gm^R^, oriR6K, pBG-derived, promoter 14c | Zobel et al. (2015) |
| pBG14d | Km^R^, Gm^R^, oriR6K, pBG-derived, promoter 14d | Zobel et al. (2015) |
| pBG14e | Km^R^, Gm^R^, oriR6K, pBG-derived, promoter 14e | Zobel et al. (2015) |
| pBG14f | Km^R^, Gm^R^, oriR6K, pBG-derived, promoter 14f | Zobel et al. (2015) |
| pBG14g | Km^R^, Gm^R^, oriR6K, pBG-derived, promoter 14g | Zobel et al. (2015) |
| pBG14f_##_14g | Km^R^, Gm^R^, oriR6K, pBG-derived, stacked promoter 14f/14g, spacer with varying length from ten to 100 bp | This work |
| pBG_80i | Km^R^, Gm^R^, oriR6K, pBG-derived, promoter-less control, reverse complement spacer sequence with 80 bp length | This work |
| pBG_80new | Km^R^, Gm^R^, oriR6K, pBG-derived, promoter-less control, new spacer sequence with 80 bp length | This work |
| pBG14f_80i_14g | Km^R^, Gm^R^, oriR6K, pBG-derived, stacked promoter 14f/14g, inverted spacer with a length of 80 bp | This work |
| pBG14f_80new_14g | Km^R^, Gm^R^, oriR6K, pBG-derived, stacked promoter 14f/14g, new spacer sequence with 80 bp length | This work |
| pBG14a_80i_14g | Km^R^, Gm^R^, oriR6K, pBG-derived, stacked promoter 14a/14g, inverted spacer with a length of 80 bp | This work |
| pBG14b_80i_14g | Km^R^, Gm^R^, oriR6K, pBG-derived, stacked promoter 14b/14g, inverted spacer with a length of 80 bp | This work |
| pBG14c_80i_14g | Km^R^, Gm^R^, oriR6K, pBG-derived, stacked promoter 14c/14g, inverted spacer with a length of 80 bp | This work |
| pBG14d_80i_14g | Km^R^, Gm^R^, oriR6K, pBG-derived, stacked promoter 14d/14g, inverted spacer with a length of 80 bp | This work |
| pBG14e_80i_14g | Km^R^, Gm^R^, oriR6K, pBG-derived, stacked promoter 14e/14g, inverted spacer with a length of 80 bp | This work |
| pBG14f_80i_14g | Km^R^, Gm^R^, oriR6K, pBG-derived, stacked promoter 14f_80i_14g, inverted spacer with a length of 80 bp | This work |
| pBG14g_80i_14g | Km^R^, Gm^R^, oriR6K, pBG-derived, stacked promoter 14g_80i_14g, inverted spacer with a length of 80 bp | This work |
| pBG14f_80i_14f_80i_14g | Km^R^, Gm^R^, oriR6K, pBG-derived, stacked promoter 14f_80i_14f_80i_14g, inverted spacer with a length of 80 bp | This work |
| pBG14g_80i_14a | Km^R^, Gm^R^, oriR6K, pBG-derived, stacked promoter 14g_80i_14a, inverted spacer with a length of 80 bp | This work |
| pBG14a_80i | Km^R^, Gm^R^, oriR6K, pBG-derived, first position promoter control 14a, inverted spacer with a length of 80 bp | This work |
| pBG14b_80i | Km^R^, Gm^R^, oriR6K, pBG-derived, first position promoter control 14b, inverted spacer with a length of 80 bp | This work |
| pBG14c_80i | Km^R^, Gm^R^, oriR6K, pBG-derived, first position promoter control 14c, inverted spacer with a length of 80 bp | This work |
| pBG14d_80i | Km^R^, Gm^R^, oriR6K, pBG-derived, first position promoter control 14d, inverted spacer with a length of 80 bp | This work |
| pBG14e_80i | Km^R^, Gm^R^, oriR6K, pBG-derived, first position promoter control 14e, inverted spacer with a length of 80 bp | This work |
| pBG14f_80i | Km^R^, Gm^R^, oriR6K, pBG-derived, first position promoter control 14f, inverted spacer with a length of 80 bp | This work |
| pBG14g_80i | Km^R^, Gm^R^, oriR6K, pBG-derived, first position promoter control 14g, inverted spacer with a length of 80 bp | This work |
| pBG_80i_14a | Km^R^, Gm^R^, oriR6K, pBG-derived, second position promoter control 14a, inverted spacer with a length of 80 bp | This work |
| pBG_80i_14c | Km^R^, Gm^R^, oriR6K, pBG-derived, second position promoter control 14c, inverted spacer with a length of 80 bp | This work |
| pBG_80i_14d | Km^R^, Gm^R^, oriR6K, pBG-derived, second position promoter control 14d, inverted spacer with a length of 80 bp | This work |
| pBG_80i_14e | Km^R^, Gm^R^, oriR6K, pBG-derived, second position promoter control 14e, inverted spacer with a length of 80 bp | This work |
| pBG_80i_14g | Km^R^, Gm^R^, oriR6K, pBG-derived, second position promoter control 14g, inverted spacer with a length of 80 bp | This work |
| pBG14f_80new | Km^R^, Gm^R^, oriR6K, pBG-derived, first position promoter control 14f, new spacer sequence with 80 bp length | This work |
| pBG_80bp_14g_new | Km^R^, Gm^R^, oriR6K, pBG-derived, second position promoter control 14g, new spacer sequence with 80 bp length | This work |
| pBG14g_SPL_PosZZ_N | Km^R^, Gm^R^, oriR6K, pBG-derived, single nucleotide promoter library with specific positions changes, library is based on 14g | This work |
| pBG14g_1a_80i | Km^R^, Gm^R^, oriR6K, pBG-derived, first position promoter control 14g_1a with change at first nucleotide in -35 element, inverted spacer with a length of 80 bp | This work |
| pBG14g_1c_80i | Km^R^, Gm^R^, oriR6K, pBG-derived, first position promoter control 14c_1c with change at first nucleotide in -35 element, inverted spacer with a length of 80 bp | This work |
| pBG14g_1g_80i | Km^R^, Gm^R^, oriR6K, pBG-derived, first position promoter control 14g_1g with change at first nucleotide in -35 element, inverted spacer with a length of 80 bp | This work |
| pBG14g_1t_80i | Km^R^, Gm^R^, oriR6K, pBG-derived, first position promoter control 14g_1t with change at first nucleotide in -35 element, inverted spacer with a length of 80 bp | This work |
| pBG14g_2a_80i | Km^R^, Gm^R^, oriR6K, pBG-derived, first position promoter control 14g_2a with change at second nucleotide in -35 element, inverted spacer with a length of 80 bp | This work |
| pBG14g_2c_80i | Km^R^, Gm^R^, oriR6K, pBG-derived, first position promoter control 14c_2c with change at second nucleotide in -35 element, inverted spacer with a length of 80 bp | This work |
| pBG14g_2g_80i | Km^R^, Gm^R^, oriR6K, pBG-derived, first position promoter control 14g_2g with change at second nucleotide in -35 element, inverted spacer with a length of 80 bp | This work |
| pBG14g_2t_80i | Km^R^, Gm^R^, oriR6K, pBG-derived, first position promoter control 14g_2t with change at second nucleotide in -35 element, inverted spacer with a length of 80 bp | This work |
| pBG14g_26a_80i | Km^R^, Gm^R^, oriR6K, pBG-derived, first position promoter control 14c_26a with change at third nucleotide in -10 element, inverted spacer with a length of 80 bp | This work |
| pBG14g_26c_80i | Km^R^, Gm^R^, oriR6K, pBG-derived, first position promoter control 14c_26a with change at third nucleotide in -10 element, inverted spacer with a length of 80 bp | This work |
| pBG14g_26g_80i | Km^R^, Gm^R^, oriR6K, pBG-derived, first position promoter control 14c_26a with change at third nucleotide in -10 element, inverted spacer with a length of 80 bp | This work |
| pBG14g_26t_80i | Km^R^, Gm^R^, oriR6K, pBG-derived, first position promoter control 14c_26a with change at third nucleotide in -10 element, inverted spacer with a length of 80 bp | This work |

Supplementary Table 10: Complete list of tested single nucleotide polymorphism and Zobel et al. (2015) promoter sequences. 14g represents the original sequence, whereas 14G promoters include changes indicated as lowercase letters.

| Promoter name | Core sequence 5´-3´ | Activity relative to 14g [%] |
| --- | --- | --- |
| 14g | TTGACAAGGCTCTCGCGGCCAGGTATAATT | 100±2,7 |
| 14a | TTGACATGGATATAATGTATGTA | 1±0,1 |
| 14G_3_a | TTaACAAGGCTCTCGCGGCCAGGTATAATT | 3±0,4 |
| 14G_1_c | cTGACAAGGCTCTCGCGGCCAGGTATAATT | 4±0,1 |
| 14G_1_a | aTGACAAGGCTCTCGCGGCCAGGTATAATT | 7±0,4 |
| 14G_23_t | TTGACAAGGCTCTCGCGGCCAGtTATAATT | 8±0,5 |
| 14G_5_g | TTGAgAAGGCTCTCGCGGCCAGGTATAATT | 9±0,3 |
| 14G_1_g | gTGACAAGGCTCTCGCGGCCAGGTATAATT | 14±0,6 |
| 14G_4_g | TTGgCAAGGCTCTCGCGGCCAGGTATAATT | 17±1,5 |
| 14G_2_c | TcGACAAGGCTCTCGCGGCCAGGTATAATT | 21±0,4 |
| 14b | TTGACATGCGTGATGTTTAGAATTATAATT | 23±0,4 |
| 14G_25_g | TTGACAAGGCTCTCGCGGCCAGGTgTAATT | 30±2,2 |
| 14G_2_g | TgGACAAGGCTCTCGCGGCCAGGTATAATT | 32±0,8 |
| 14c | TTGACATGTCAATTTTTATGTTGTATAATA | 35±0,5 |
| 14G_2_a | TaGACAAGGCTCTCGCGGCCAGGTATAATT | 39±0,5 |
| 14G_30_g | TTGACAAGGCTCTCGCGGCCAGGTATAATg | 42±1,1 |
| 14G_6_c | TTGACcAGGCTCTCGCGGCCAGGTATAATT | 45±1,9 |
| 14G_24_a | TTGACAAGGCTCTCGCGGCCAGGaATAATT | 45±1,9 |
| 14G_6_t | TTGACtAGGCTCTCGCGGCCAGGTATAATT | 51±2,4 |
| 14d | TTGACATCCGACATTCGCGACTGTATAATA | 53±0,8 |
| 14G_21_t | TTGACAAGGCTCTCGCGGCCtGGTATAATT | 54±1,2 |
| 14G_9_t | TTGACAAGtCTCTCGCGGCCAGGTATAATT | 54±0,5 |
| 14G_8_a | TTGACAAaGCTCTCGCGGCCAGGTATAATT | 55±0,5 |
| 14G_29_g | TTGACAAGGCTCTCGCGGCCAGGTATAAgT | 60±0,9 |
| 14G_8_t | TTGACAAtGCTCTCGCGGCCAGGTATAATT | 62±1,2 |
| 14G_20_a | TTGACAAGGCTCTCGCGGCaAGGTATAATT | 64±0,9 |
| 14G_30_a | TTGACAAGGCTCTCGCGGCCAGGTATAATa | 64±2,8 |
| 14G_19_a | TTGACAAGGCTCTCGCGGaCAGGTATAATT | 65±0,1 |
| 14G_10_a | TTGACAAGGaTCTCGCGGCCAGGTATAATT | 66±1,2 |
| 14G_16_t | TTGACAAGGCTCTCGtGGCCAGGTATAATT | 66±1,6 |
| 14G_12_a | TTGACAAGGCTaTCGCGGCCAGGTATAATT | 67±0,4 |
| 14G_12_g | TTGACAAGGCTgTCGCGGCCAGGTATAATT | 67±1,1 |
| 14G_20_g | TTGACAAGGCTCTCGCGGCgAGGTATAATT | 67±1,1 |
| 14G_14_t | TTGACAAGGCTCTtGCGGCCAGGTATAATT | 68±0,6 |
| 14G_11_g | TTGACAAGGCgCTCGCGGCCAGGTATAATT | 68±2,2 |
| 14G_12_t | TTGACAAGGCTtTCGCGGCCAGGTATAATT | 68±2,5 |
| 14G_22_t | TTGACAAGGCTCTCGCGGCCAtGTATAATT | 68±3,8 |
| 14G_13_g | TTGACAAGGCTCgCGCGGCCAGGTATAATT | 68±1,5 |
| 14G_21_c | TTGACAAGGCTCTCGCGGCCcGGTATAATT | 69±2,6 |
| 14G_22_a | TTGACAAGGCTCTCGCGGCCAaGTATAATT | 69±2,4 |
| 14G_17_c | TTGACAAGGCTCTCGCcGCCAGGTATAATT | 70±1,8 |
| 14G_10_g | TTGACAAGGgTCTCGCGGCCAGGTATAATT | 70±1 |
| 14G_21_g | TTGACAAGGCTCTCGCGGCCgGGTATAATT | 70±2 |
| 14G_17_a | TTGACAAGGCTCTCGCaGCCAGGTATAATT | 71±1,5 |
| 14G_15_t | TTGACAAGGCTCTCtCGGCCAGGTATAATT | 71±0,7 |
| 14G_16_a | TTGACAAGGCTCTCGaGGCCAGGTATAATT | 72±2,2 |
| 14G_7_t | TTGACAtGGCTCTCGCGGCCAGGTATAATT | 74±2,8 |
| 14G_14_g | TTGACAAGGCTCTgGCGGCCAGGTATAATT | 75±3 |
| 14G_28_t | TTGACAAGGCTCTCGCGGCCAGGTATAtTT | 81±0,5 |
| 14G_28_g | TTGACAAGGCTCTCGCGGCCAGGTATAgTT | 83±0,9 |
| 14G_27_g | TTGACAAGGCTCTCGCGGCCAGGTATgATT | 84±0,9 |
| 14G_26_a | TTGACAAGGCTCTCGCGGCCAGGTAaAATT | 84±1,4 |
| 14G_28_c | TTGACAAGGCTCTCGCGGCCAGGTATAcTT | 84±4,3 |
| 14G_26_c | TTGACAAGGCTCTCGCGGCCAGGTAcAATT | 89±1,3 |
| 14e | TTGACAACACTCGAAAAGCCGAGTATAATC | 91±1,4 |
| 14f | TTGACATGACATGGTTTTGAGGGTATAATG | 93±2,6 |

## Supplementary Figures

Supplementary Figure 1: Characterized stacking promoters and controls genomically integrated in *P. putida* KT2440. Promoter 14f/14f/14g was accidentally generated during cloning procedures for combination 14f_80i_14g. Triple variance has an additional 14f_80i sequence inserted, whereat two additional nucleotides are integrated in the middle 14f promoter and one nucleotide is missing in the first 80 base pair spacer. All strains were cultured in a BioLector in minimal medium with 20 mM glucose in a 96 well plate. Identical strains from at least two from different transformations were tested, with three biological replicates each. Error bars indicate the standard error of the mean (n>6).
